# The efficacy of articaine in pain management during endodontic procedures in pediatric patients

**DOI:** 10.1186/s13741-024-00389-5

**Published:** 2024-04-30

**Authors:** Yilei Che, Minhua Wang, Xiaozhen Wu, Xueling Wang

**Affiliations:** 1 Department of Stomatology, Aerospace Center Hospital (ASCH), No.15, YuQuan Road, Haidian District, Beijing, 100049 China; 2 Department of Stomatology, Eye Hospital China Academy of Chinese Medical Sciences, Beijing, 100040 China

**Keywords:** Articaine, Children, Lidocaine, Dental pulp disease, Blood pressure, Painless treatment

## Abstract

**Objective:**

This trial aimed to study the efficacy of articaine in pain management during endodontic procedures in pediatric patients.

**Methods:**

Ninety-eight children who received endodontic painless treatment were collected and randomly divided into the control group and observation group, with 49 cases in each group. The control group received infiltration anesthesia with lidocaine, and the observation group received infiltration anesthesia with articaine. Anesthesia effect, anesthesia onset time, sensory recovery time, duration of anesthesia, pain intensity, blood pressure, heart rate, and adverse reactions were compared.

**Results:**

The effective rate of anesthesia in the observation group was higher than that in the control group. The anesthesia onset time and sensory recovery time were shorter, the duration of anesthesia was longer, and the VAS score and facial expression score were lower in the observation group than in the control group. The heart rate of the observation group was lower, and diastolic blood pressure was higher than those of the control group. The total incidence of adverse reactions in the observation group was lower than that in the control group.

**Conclusion:**

In the treatment of dental pulp diseases in children, the use of articaine can achieve better anesthesia effect and rapid onset of anesthesia and has less impact on the patient’s blood pressure and heart rate, but it also can relieve pain and has good safety after the use of medication. It is worthy of clinical application.

## Introduction

Throughout the world, endodontic disease, a biofilm infection of the root canal space, is one of the most common causes of dental morbidity (Abusrewil et al. 2020). Endodontic disease is located in a rigid chamber that provides strong mechanical support and protection from the microbe-rich oral environment. Whenever the dental pulp loses its structural integrity, it is susceptible to irritations from the mouth. This disease can lead to progressive lesions of dental hard tissues, purulent necrosis of pulp tissue, and severe toothache (Kratunova and Silva 2018). Dental pulp disease is one of the most common oral diseases, although functional pulp regeneration remains challenging (Zheng et al. 2023). Surgery in endodontics has been refined as a result of a greater understanding of endodontic disease and treatment failures. Surgical armamentarium and technique advancements have also improved the endodontic surgical outcome with the introduction of newer materials and techniques (Chong and Rhodes 2014). However, clinical treatment of endodontic disease is usually based on pulp opening or pulp extraction, and children need to bear greater pain during surgery, which is difficult to cooperate well with treatment and affects the overall progress of surgery. The importance of reasonable measures of anesthesia for children cannot be ignored.

As anesthesia technology continues to improve, endodontic painless treatments have become more widespread in dentistry. The main method of pain control in dentistry is local anesthesia with lidocaine and articaine (Nagendrababu et al. 2020; St George, et al. 2018). Indeed, great anesthetic effects can reduce pain and discomfort, improve patient cooperation, and reduce anxiety (Mathison et al. 2023). As an antiarrhythmic agent, lidocaine is an amide local anesthetic initially administered intravenously (Beaussier et al. 2018). Articaine is a unique amide compound that contains a thiophene ring and an additional ester group (Yapp et al. 2011). It has been practiced that lidocaine local analgesia for pain management helps homeostasis and stabilizes vital signs during pediatric dental rehabilitation (Batawi 2013), and articaine can control pain while reducing the dose given in children (Leith et al. 2012). Studies have evaluated and compared the efficacy of lidocaine and articaine in children populations (Bonifacio 2018; Bartlett and Mansoor 2016), and it has been recognized that in terms of pulpal anesthesia, articaine is superior to lidocaine (Powell 2012). Therefore, in this paper, the anesthetic advantages of articaine were discussed by comparing the anesthetic effects of articaine and lidocaine, hoping to provide a reference for the clinical treatment of dental pulp diseases in children. The innovation this study was that we, for the first time, investigated the efficacy of articaine in pain management during endodontic procedures in pediatric patients.

## Materials and methods

### Ethics statement

This study was approved by the Ethics Committee of Aerospace Center Hospital (approval number: 20190922). Family members signed the informed consent form.

### Participants

**Fig. 1:**
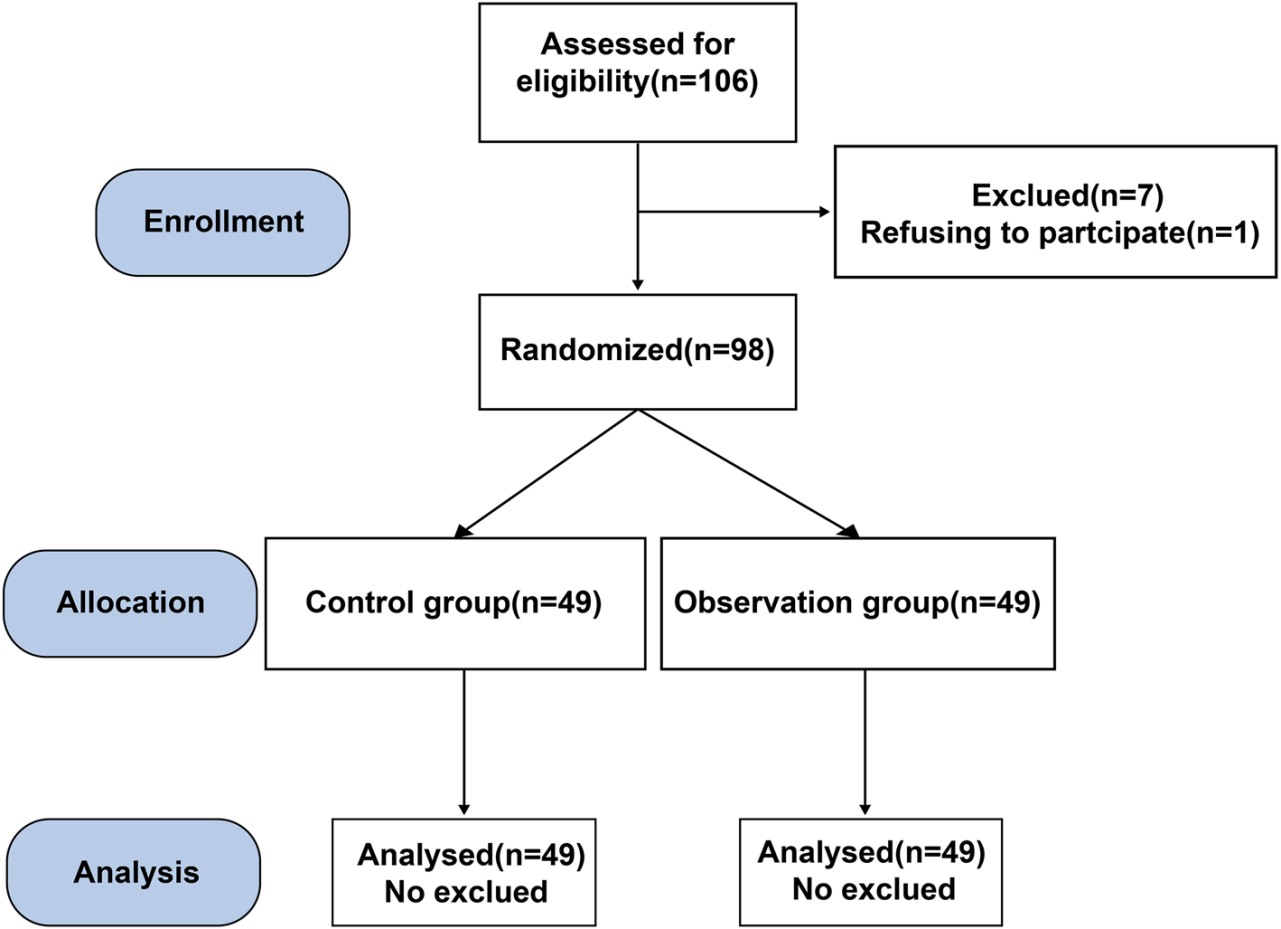
The participant flow diagram

This was a prospective study. Children with dental pulp diseases admitted to Aerospace Center Hospital from January 2020 to December 2020 were selected as study subjects. The inclusion criteria are as follows: (1) children met the diagnostic criteria for dental pulp disease in accordance with the Guidelines for radiographic examination in cariology and endodontics (Society of, C. and C.S.A. Endodontics 2021); (2) children aged 6–15 years old; (3) children received treatment for the first time; (4) children had dental pulp disease in a single tooth; (5) children with certain understanding ability and normal communication ability; (6) family members signed the informed consent form. The exclusion criteria are as follows: (1) children with blood system diseases; (2) children with severe immune system disease; (3) children who were allergic or allergic to the study drug; (4) children with serious infectious diseases; (5) children with incomplete information. After inclusion and exclusion criteria, 98 children were ultimately enrolled in this study. These 98 eligible participants randomly assigned in a 1:1 ratio to the control group and observation group using computer-generated randomization numbers in sealed opaque envelopes prepared by an individual not involved in the study. The control group was anesthetized with lidocaine, while the observation group was anesthetized with articaine. The participant flow diagram is shown in Fig. 1.

### Methods of anesthesia

The children in the control group were anesthetized with lidocaine hydrochloride injection (Shandong Hualu Pharmaceutical Co., Ltd., Shandong, China; No. H37022108; specifications: 20 ml: 0.4 g). Injections were performed on the buccal and labial sides of the affected teeth, with a dosage of 2.2 ml for each injection point of the posterior teeth and 1.1 ml for each injection point of the anterior teeth, and after 6 min of injections, the condition of the affected children was observed, and endodontic procedures were performed.

The children in the observation group were anesthetized with Compound Articaine Hydrochloride Injection (Ma’anshan Fengyuan Pharmaceutical Co., Ltd., Anhui, China; No. H20045881; specifications: 1.7 ml: 68 mg of articaine hydrochloride, 17 μg of epinephrine), which was injected in the submucosal, subperiosteal, and periodontal ligament locations of the affected teeth, with 1.6 ml of injection dose per injection site for the posterior teeth, and 0.7 ml per injection site for the anterior teeth, and after 6 min of injection, the condition of the affected children was observed and endodontic procedures were performed.

### Observation indices

The effect of anesthesia, onset time to anesthesia, pain intensity, heart rate and blood pressure level, and adverse effects were evaluated in both groups.

1. Anesthetic effect was compared. Obviously effective: no pain during treatment and smooth treatment process; effective: dental pain appeared but could be tolerated, and the treatment was successfully completed; ineffective: obvious pain in the course of treatment and could not be tolerated, and treatment was even stopped. Total effective rate = (obviously effective + effective)/49 × 100%
2. Anesthesia onset time, anesthesia duration, and sensory recovery time were compared
3. Pain intensity: the visual analog scale (VAS) (VAS could be used for children over 6 years old (Sardar et al. 2017; May et al. 2018)) was utilized to assess the pain level of the children. The total score of the scale was 0–10 points, in which 0 was no pain; below 3 was mild pain, which was tolerated by the child; 4–6 was pain that interfered with sleep, which was still tolerated; 7–10 was intense pain, which was usually difficult to tolerate and interfered with appetite and sleep. The higher the score, the more intense the pain. Kuttner’s facial expression score was adopted to assess the comfort level of the patients with the anesthetic injection, which was quantified by a number from 0 to 6, with a higher number indicating a lower comfort level, where 0 represented comfort, and 6 represented the patient’s facial expression of pain
4. Heart rate and blood pressure levels: heart rate, systolic blood pressure (SBP), and diastolic blood pressure (DBP) before and after anesthesia were monitored by an automatic electronic sphygmomanometer
5. Adverse reactions, including dizziness, headache, tachycardia, nausea, and vomiting, were recorded

### Statistical analysis

Blind methods were used for data monitors and statistical analysts. Data were evaluated by the SPSS26.0 statistical software (IBM Corp., Armonk, NY, USA). Enumeration data expressed as [cases (%)] were statistically compared by chi-squared test or Fisher’s exact test. Normally distributed data which are presented as the mean ± standard deviation were compared by *t*-test. *P* < 0.05 indicated a significant difference.

## Results

### General data

Age, gender, course of disease, and onset site of the teeth did not differ significantly between the observation and control groups (*P* > 0.05), indicating comparability (Table 1).

**Table 1:** Comparison of general information between the two groups

Parameters	Control group (*n* = 49)	Observation group (*n* = 49)	*p* value
Age (years)	9.49 ± 2.14	9.51 ± 2.28	0.964
Gender			0.686
Male	24	27	
Female	25	22	
Course of disease (days)	4.57 ± 1.71	4.80 ± 1.27	0.463
Onset site of the teeth			0.840
Anterior teeth	24	23	
Posterior teeth	25	26	

### Analysis of anesthetic effect

The effective rate of anesthesia in the observation group was 95.92% (47/49) higher than that in the control group 77.55% (38/49) (*P* < 0.05, Table 2).

**Table 2:** Analysis of anesthetic effects in two groups (cases/%)

Groups	Obviously effective	Effective	Ineffective	Total effective rate	*p* value
Control group (*n* = 49)	15 (30.61%)	23 (46.94%)	11 (22.45%)	38 (77.55%)	0.007
Observation group (*n* = 49)	21 (42.86%)	26 (53.06%)	2 (4.08%)	47 (95.92%)	

### Anesthesia onset time, duration of anesthesia, and time of sensory recovery

The onset time and sensory recovery time of anesthesia in the observation group were shorter than those in the control group, while the duration of anesthesia was longer (*P* < 0.05, Table 3).

**Table 3:** Comparison of anesthesia onset time, duration of anesthesia, and sensory recovery time (min) between the two groups

Groups	Anesthesia onset time	Duration of anesthesia	Sensory recovery time
Control group (*n* = 49)	4.63 ± 0.57	146.12 ± 12.63	171.51 ± 18.48
Observation group (*n* = 49)	2.55 ± 0.54	157.61 ± 19.26	164.57 ± 12.42
*p* value	< 0.001	< 0.001	0.032

### VAS scores and facial expression scores

VAS scores and facial expression scores in the observation group were lower than those in the control group (*P* < 0.05, Table 4).

**Table 4:** Comparison of VAS scores and facial expression scores between the two groups

Groups	VAS scores	Facial expression scores
Control group (*n* = 49)	2.63 ± 0.57	1.61 ± 0.50
Observation group (*n* = 49)	1.55 ± 0.54	1.25 ± 0.37
*p* value	< 0.001	< 0.001

### Blood pressure and heart rate

Before anesthesia, heart rate, SBP, and DBP were not significantly different between the observation and control groups two groups (*P* > 0.05). After anesthesia, as compared to the control group, the observation group had a lower heart rate and a higher DBP (*P* < 0.05), and when comparing the SBP of patients in both groups, the difference was not statistically significant (*P* > 0.05) (Table 5).

**Table 5:** Comparison of blood pressure and heart rate between the two groups

Groups	Diastolic blood pressure (mmHg)		Systolic blood pressure (mmHg)		Heart rate (beats/min)	
	Before anesthesia	After anesthesia	Before anesthesia	After anesthesia	Before anesthesia	After anesthesia
Control group (*n* = 49)	74.69 ± 8.67	70.08 ± 8.06	117.57 ± 11.57	115.55 ± 11.52	75.61 ± 7.28	81.35 ± 8.60
Observation group (*n* = 49)	75.14 ± 8.51	74.31 ± 7.20	118.47 ± 12.22	117.41 ± 11.10	75.10 ± 7.19	76.43 ± 6.57
*p* value	0.796	0.007	0.710	0.418	0.728	0.002

### Adverse reaction rate

The total incidence of dizziness, headache, tachycardia, nausea, and vomiting in the observation group (4.08%) was significantly lower than that in the control group (20.41%) (*P* < 0.05, Table 6).

**Table 6:** Comparison of adverse reaction rate between two groups (cases/%)

Groups	Dizziness	Headache	Tachycardia	Nausea	Vomiting	Total adverse reactions	*p* value
Control group (*n* = 49)	2 (4.08%)	2 (4.08%)	1 (2.04%)	3 (6.12%)	2 (4.08%)	10 (20.41%)	0.028
Observation group (*n* = 49)	1 (2.04%)	1 (2.04%)	0	0	0	2 (4.08%)	

## Discussion

Endodontic disease refers to an inflammatory condition of the pulp (pulpitis) or necrosis of the pulp (partial or complete) (Niemiec 2005). Endodontic disease is mainly caused by progressive lesions of dental hard tissue in children, which can produce obvious pain and affect children’s health. After the onset of endodontic disease, severe spontaneous pain will aggravate with oral caloric stimulation. As a special group of patients, children have a low tolerance to pain and poor compliance and even have anxiety and fear in severe cases. In this regard, painless treatment is the core of improving the treatment compliance of children and thereafter ensuring the success of treatment. Therefore, it is of great significance to select safe and effective anesthesia drugs to alleviate pain, improve treatment compliance, and ensure successful completion of surgical treatment.

In this study, lidocaine administrated by children in the control group is usually used as a local anesthetic drug in clinical practice, with strong and durable anesthetic effect and obvious bidirectional excitatory and inhibitory effects on the central nervous system (Hermanns et al. 2019). Lidocaine has anti-inflammatory and opiate-sparing properties, a combination of characteristics which results in an array of effects, including a reduction in postoperative pain and opiate consumption as well as a reduction in the duration of digestive ileus (Beaussier et al. 2018). However, after local anesthesia with lidocaine, the side effects also increase with the increase of dose (Bahar and Yoon 2021). Lidocaine may induce fear or anxiety-related adverse reactions, psychogenic effects, and allergic reactions (Anderson, et al. 1990). Patients in the observation group were anesthetized with articaine. The molecular structure of articaine is featured with both lipophilic and hydrophilic ends connected by a hydrocarbon chain, and the “CO linkage” between the lipophilic aromatic ring and the hydrocarbon chain classifies articaine as an ester local anesthetic, and the link is metabolized by plasma cholinesterase. Next, articaine is rapidly metabolized to its inactive metabolite articaine acid through hydrolysis, which is partially metabolized to articainic acid glucuronide in the kidneys (Vree and Gielen 2005). Articaine has a unique lipophilicity switch in terms of its capability to form an intramolecular hydrogen bond. This intramolecular hydrogen bond is a new and additional solvent-dependent mechanism that can modulate the lipophilicity of articaine, thereby enhancing its diffusion through membranes and connective tissue (Skjevik et al. 2011). The results showed that the total effective rate of anesthesia in the observation group was higher, anesthesia onset time and sensory recovery time were shorter, and duration of anesthesia was longer than those in the control group, revealing that articaine can improve the anesthesia effect and effectively shorten the onset time of anesthesia. Currently, studies have illustrated that local anesthesia with articaine is more effective than lidocaine for dentistry treatment (Nagendrababu et al. 2020; Khan, et al. 2021; Taneja et al. 2020; Martin et al. 2021). It is also noted that for supplementary infiltration after mandibular block anesthesia, articaine has a significant advantage over lidocaine in patients with symptomatic irreversible pulpitis (Kung et al. 2015). Articaine has been revealed to enhance higher anesthesia success and longer duration of anesthesia in comparison to lidocaine for most of the teeth after incisive/mental nerve block (Batista da Silva, et al. 2010). Moreover, articaine is superior to lidocaine for its application in lower third molar surgeries because of the shorter time until the onset of action, higher success rate, and greater control of intraoperative and postoperative pain as well as longer duration of the anesthetic effect (Nogueira et al. 2023).

Moreover, the VAS score was lower and the onset time of anesthesia was shorter than that in the control group, indicating that articaine can reduce pain during surgery. Articaine contains a thiophene group, which increases its liposolubility. Since articaine diffuses better through soft tissues than other anesthetics, a higher concentration of anesthetic is delivered, more longitudinal spread is achieved, and conduction is effectively blocked (Potocnik et al. 2006). It has been reported that articaine is more effective than lidocaine in reducing self-reported pain after dental treatment, although there is no difference between lidocaine and articaine during treatment (Bonifacio 2018; Tong et al. 2018). Measurements of heart rate and blood pressure are used as physiological parameters because they provide indirect measures of anxiety and pain. The body’s response to stressful situations or painful stimuli can be seen as higher heart rate levels and higher SBP values (Rathi et al. 2019). In this trial, after anesthesia, as compared to the control group, the observation group had a lower heart rate and a higher DBP. This shows that articaine effectively relieved negative emotion and pain of children in the treatment. Furthermore, it was recorded that the total incidence of adverse reactions in the observation group was significantly lower than that in the control group, suggesting that while achieving the anesthetic effect, the adverse reactions caused by articaine are less and the clinical safety is higher. Consistently, a comparison study has measured a lower incidence of adverse events in the management of tooth pulp disease after articaine anesthesia than lidocaine anesthesia (Li, et al. 2018).

In summary, the advantages of articaine applied in the painless treatment of dental pulp disease in children are as follows: (1) the anesthetic effect is satisfactory, which can improve the cooperation of children in the treatment process and reduce the difficulty of treatment; (2) the effect is fast, which can effectively alleviate the negative psychology and improve treatment compliance; (3) the incidence of complications can be significantly reduced. However, we did not do the sample size calculations, and more clinical studies are needed to further confirm the authenticity of the results.

## Data Availability

The data that support the findings of this study are available from the corresponding author upon reasonable request.

## References

[CR1] Abusrewil S (2020). Detection, treatment and prevention of endodontic biofilm infections: what’s new in 2020?. Crit Rev Microbiol.

[CR2] Anderson KP (1990). Conduction velocity depression and drug-induced ventricular tachyarrhythmias. Effects of lidocaine in the intact canine heart. Circulation.

[CR3] Bahar E, Yoon H (2021). Lidocaine: a local anesthetic, its adverse effects and management. Medicina (Kaunas).

[CR4] Bartlett G, Mansoor J (2016). Articaine buccal infiltration vs lidocaine inferior dental block - a review of the literature. Br Dent J.

[CR5] Beaussier M (2018). Perioperative use of intravenous lidocaine. Drugs.

[CR6] Bonifacio CC (2018). The efficacy of articaine and lidocaine local anaesthetic in child patients. Evid Based Dent.

[CR7] Chong BS, Rhodes JS (2014). Endodontic surgery. Br Dent J.

[CR8] da BatistaSilva C (2010). Anesthetic efficacy of articaine and lidocaine for incisive/mental nerve block. J Endod.

[CR9] El Batawi HY (2013). Lidocaine use for pain management during paediatric dental rehabilitation under general anaesthesia. Eur Arch Paediatr Dent.

[CR10] Hermanns H (2019). Molecular mechanisms of action of systemic lidocaine in acute and chronic pain: a narrative review. Br J Anaesth.

[CR11] Khan Q (2021). Comparison of anaesthetic efficacy of articaine and lidocaine in nonsurgical endodontic treatment of permanent mandibular molars with symptomatic irreversible pulpitis. A randomized clinical trial. J Ayub Med Coll Abbottabad.

[CR12] Kratunova E, Silva D (2018). Pulp therapy for primary and immature permanent teeth: an overview. Gen Dent.

[CR13] Kung J, McDonagh M, Sedgley CM (2015). Does articaine provide an advantage over lidocaine in patients with symptomatic irreversible pulpitis? A systematic review and meta-analysis. J Endod.

[CR14] Le May S (2018). Comparison of the psychometric properties of 3 pain scales used in the pediatric emergency department: visual analogue scale, faces pain scale-revised, and colour analogue scale. Pain.

[CR15] Leith R, Lynch K, O’Connell AC (2012). Articaine use in children: a review. Eur Arch Paediatr Dent.

[CR16] Li J (2018). Comparison of clinical efficacy and safety between articaine and lidocaine in the anaesthesia management of tooth pulp disease. Pak J Pharm Sci.

[CR17] Martin E (2021). Articaine in dentistry: an overview of the evidence and meta-analysis of the latest randomised controlled trials on articaine safety and efficacy compared to lidocaine for routine dental treatment. BDJ Open.

[CR18] Mathison, M. and T. Pepper, Local anesthesia techniques in dentistry and oral surgery, in StatPearls. Treasure Island (FL) ineligible companies. Disclosure: Tom Pepper declares no relevant financial relationships with ineligible companies. 2023 35593805

[CR19] Nagendrababu V (2020). Is articaine more effective than lidocaine in patients with irreversible pulpitis?. An Umbrella Review Int Endod J.

[CR20] Niemiec BA (2005). Fundamentals of endodontics. Vet Clin North Am Small Anim Pract.

[CR21] Nogueira EC (2023). Why choose articaine over lidocaine for the removal of third molars? Systematic review and meta-analysis. J Clin Exp Dent.

[CR22] Potocnik I (2006). Articaine is more effective than lidocaine or mepivacaine in rat sensory nerve conduction block in vitro. J Dent Res.

[CR23] Powell V (2012). Articaine is superior to lidocaine in providing pulpal anesthesia. J Am Dent Assoc.

[CR24] Rathi NV (2019). Anesthetic efficacy of buccal infiltration articaine versus lidocaine for extraction of primary molar teeth. Anesth Prog.

[CR25] Sardar A (2017). Comparison of efficacy of oral versus regional clonidine for postoperative analgesia following ilioinguinal/iliohypogastric block in children: a prospective, randomized, double-blinded, placebo-controlled study. Anesth Essays Res.

[CR26] Skjevik AA (2011). Intramolecular hydrogen bonding in articaine can be related to superior bone tissue penetration: a molecular dynamics study. Biophys Chem.

[CR27] Society of, C. and C.S.A. Endodontics (2021). Guidelines for radiographic examination in cariology and endodontics. Zhonghua Kou Qiang Yi Xue Za Zhi.

[CR28] St George G (2018). Injectable local anaesthetic agents for dental anaesthesia. Cochrane Database Syst Rev.

[CR29] Taneja S, Singh A, Jain A (2020). Anesthetic effectiveness of articaine and lidocaine in pediatric patients during dental procedures: a systematic review and meta-analysis. Pediatr Dent.

[CR30] Tong HJ (2018). Anaesthetic efficacy of articaine versus lidocaine in children’s dentistry: a systematic review and meta-analysis. Int J Paediatr Dent.

[CR31] Vree TB, Gielen MJ (2005). Clinical pharmacology and the use of articaine for local and regional anaesthesia. Best Pract Res Clin Anaesthesiol.

[CR32] Yapp KE, Hopcraft MS, Parashos P (2011). Articaine: a review of the literature. Br Dent J.

[CR33] Zheng L (2023). Injectable decellularized dental pulp matrix-functionalized hydrogel microspheres for endodontic regeneration. Acta Biomater.

